# The E3 ubiquitin ligase EDD is an adverse prognostic factor for serous epithelial ovarian cancer and modulates cisplatin resistance *in vitro*

**DOI:** 10.1038/sj.bjc.6604281

**Published:** 2008-03-18

**Authors:** P M O'Brien, M J Davies, J P Scurry, A N Smith, C A Barton, M J Henderson, D N Saunders, B S Gloss, K I Patterson, J L Clancy, V A Heinzelmann-Schwarz, R A Scolyer, Y Zeng, E D Williams, L Scurr, A DeFazio, D I Quinn, C K W Watts, N F Hacker, S M Henshall, R L Sutherland

**Affiliations:** 1 Cancer Research Program, Garvan Institute of Medical Research, Darlinghurst, Sydney, New South Wales 2010, Australia; 2 St Vincent's Clinical School, University of NSW, Sydney, New South Wales, Australia; 3 South East Area Laboratory Service, Prince of Wales Hospital, Randwick, Sydney, New South Wales 2031, Australia; 4 Division of Gynecology, University Hospital Zurich, 8091 Zurich, Switzerland; 5 Department of Anatomical Pathology, Royal Prince Alfred Hospital, Camperdown, Sydney, New South Wales 2050, Australia; 6 Centre for Cancer Research, Monash Institute of Medical Research, Monash University, Clayton, Melbourne, Victoria 3168, Australia; 7 Westmead Institute for Cancer Research, The University of Sydney at Westmead Millennium Institute, Westmead, Sydney, New South Wales 2145, Australia; 8 Department of Gynaecological Oncology, Westmead Hospital, Westmead, Sydney, New South Wales 2145, Australia; 9 Keck School of Medicine, Norris Comprehensive Cancer Center, University of Southern California, Los Angeles 90089, CA, USA; 10 Gynaecological Cancer Centre, Royal Hospital for Women, Randwick, Sydney, New South Wales 2031, Australia; 11 School of Women's and Children's Health, University of NSW, Sydney, New South Wales, Australia

**Keywords:** ovarian cancer, serous, EDD, recurrence, chemoresistance, cisplatin

## Abstract

Despite a high initial response rate to first-line platinum/paclitaxel chemotherapy, most women with epithelial ovarian cancer relapse with recurrent disease that becomes refractory to further cytotoxic treatment. We have previously shown that the E3 ubiquitin ligase, *EDD*, a regulator of DNA damage responses, is amplified and overexpressed in serous ovarian carcinoma. Given that DNA damage pathways are linked to platinum resistance, the aim of this study was to determine if EDD expression was associated with disease recurrence and platinum sensitivity in serous ovarian cancer. High nuclear EDD expression, as determined by immunohistochemistry in a cohort of 151 women with serous ovarian carcinoma, was associated with an approximately two-fold increased risk of disease recurrence and death in patients who initially responded to first-line chemotherapy, independently of disease stage and suboptimal debulking. Although EDD expression was not directly correlated with relative cisplatin sensitivity of ovarian cancer cell lines, sensitivity to cisplatin was partially restored in platinum-resistant A2780-cp70 ovarian cancer cells following siRNA-mediated knockdown of EDD expression. These results identify EDD as a new independent prognostic marker for outcome in serous ovarian cancer, and suggest that pathways involving EDD, including DNA damage responses, may represent new therapeutic targets for chemoresistant ovarian cancer.

In developed countries, epithelial ovarian cancer is the leading cause of death from gynaecological malignancies (Ries *et al*, 2007). Typically, this cancer has an insidious onset and the majority of women present with disease that has spread beyond the ovary. Although a complete clinical response to optimal surgery and first-line chemotherapy (currently usually a platinum agent in combination with paclitaxel) is frequently achieved, the majority of women will relapse with recurrent disease that becomes refractory to further cytotoxic treatment and is essentially incurable. Understanding the mechanisms that underlie the emergence of drug resistance is critical for the development of novel treatments that can prevent or overcome drug resistance and thus improve survival (Vasey, 2003; Agarwal and Kaye, 2003).

The progestin-regulated HECT/E3 ubiquitin ligase *EDD* (E3 ligase identified by differential display; also called *DD5*, *hHYD*) is the human orthologue of the *Drosophila melanogaster* ‘hyperplastic discs’ tumour suppressor gene (Mansfield *et al*, 1994). Like *hyd*, *EDD* encodes a large protein of approximately 300 kDa that contains a number of potential functional domains, including a conserved carboxy terminus HECT domain with E3 ubiquitin protein ligase activity (Callaghan *et al*, 1998). Mutagenesis studies have revealed a critical role for *hyd* in the control of cell proliferation during *Drosophila* development (Mansfield *et al*, 1994), suggesting that *hyd* functions as a tumour suppressor gene. There is now substantial evidence that EDD is most likely involved in the regulation of mammalian cell proliferation and human tumorigenesis. First, ubiquitin-mediated proteolysis is essential for the regulation of many key mammalian cellular pathways, including control of cell cycle progression, transcriptional control and DNA damage responses. Cancer-associated proteins identified as being targeted by ubiquitinylation include tumour suppressors such as p53, growth factor receptors, cell cycle regulators and transcription factors (Burger and Seth, 2004). Moreover, there are examples of E3 ligases themselves functioning as oncogenes (Daujat *et al*, 2001) or tumour suppressor genes, including the breast and ovarian cancer susceptibility gene *BRCA1* (Hashizume *et al*, 2001). HECT/E3 ligases can also function as steroid receptor transcriptional co-activators (White and Parker, 1998), with a proposed functional link to oncogenesis and hormone resistance. We have previously shown that EDD has a role in progestin signalling, being regulated both by progestin (Callaghan *et al*, 1998) and enhancing its transcriptional activity via a direct interaction between EDD and the progestin receptor (Henderson *et al*, 2002). We have also shown that EDD is a substrate of the protein kinase ERK2, a component of the mitogen-activated protein kinase pathway, critical to the regulation of many cellular processes, including proliferation, differentiation, gene transcription and cellular migration (Eblen *et al*, 2003). ERK can phosphorylate EDD both *in vitro* and *in vivo*; however, the phosphorylation sites in the EDD protein, likely to be numerous given its large size, remain to be determined. Finally, results from *EDD* knockout (*Edd*^Δ/Δ^) mice have determined that EDD plays an essential role in vascular development and angiogenesis (Saunders *et al*, 2004).

Recent evidence from our laboratory has shown that EDD is also involved in DNA damage signalling: it interacts with both the DNA-dependent protein kinase-interacting protein CIB (Henderson *et al*, 2002) and the cell checkpoint kinase CHK2, a critical mediator of the cell cycle G2/M checkpoint in response to DNA damage (Henderson *et al*, 2006), and is necessary for S-phase and G2/M checkpoint proficiency following DNA damage (Munoz *et al*, 2007). To date, there is no evidence that these proteins are ubiquitinylation substrates of EDD. Others have identified topoisomerase II*β*-binding protein, required for cell survival after DNA damage (Yamane *et al*, 2002) and associated with an increased risk of familial breast and ovarian cancer (Karppinen *et al*, 2006), as a ubiquitinylation target of EDD (Honda *et al*, 2002).

We have previously shown that the *EDD* gene locus located at 8q22.3 exhibits a high frequency of allelic imbalance in a variety of human cancers, including breast, liver and ovarian carcinoma (Clancy *et al*, 2003). At least in breast and ovarian cancer, this appears to be due to amplification, as demonstrated by EDD overexpression in these tumours. Specifically, we demonstrated that 47% of epithelial ovarian cancers exhibited allelic imbalance at the *EDD* locus, which was rarely observed in benign and borderline (low malignant potential) ovarian tumours and hence appears to be restricted to malignant disease. The highest frequency of allelic imbalance (73%) was found in serous ovarian carcinoma, the most common histological subtype of ovarian cancer, which corresponded to EDD expression in almost all of the cancers examined, as determined by immunohistochemistry (IHC) (Clancy *et al*, 2003).

The aim of the current study was therefore to determine any associations between EDD overexpression and patient outcome, in particular disease recurrence in patients with serous ovarian cancer, by correlating protein expression as measured by IHC to clinicopathological parameters in a retrospective cohort of 151 primary cancers. In addition, given that the development of resistance to platinum agents is linked to genes involved in DNA damage response pathways (Richardson and Kaye, 2005), one of the known functions of EDD, we also determined if EDD expression was associated with sensitivity to cisplatin in serous ovarian cancer.

## MATERIALS AND METHODS

### Tissue and clinicopathological data

Formalin-fixed, paraffin-embedded tissue specimens from 151 serous epithelial ovarian carcinomas were collected retrospectively from patients undergoing primary laparatomy at the Gynaecological Cancer Centre, Royal Hospital for Women between 1989 and 2002 following informed consent. Clinical (histopathologic diagnosis, treatment, surgical debulking), pathologic (tumour grade, stage) and outcome (disease recurrence, death) data on each patient were collected (Table 1). All experimental procedures were approved by the Human Research Ethics Committee of the Sydney South East Area Hospital Service, Eastern Section (00/115).

### Immunohistochemistry

Tissue core biopsies of a minimum diameter of 1.0 mm were incorporated into medium-density tissue microarrays and diagnosis confirmed by two gynaecological pathologists (JPS and RAS) as described previously (Bali *et al*, 2004; Heinzelmann-Schwarz *et al*, 2004). Tissue sectioning and IHC was performed as previously described using a rabbit anti-EDD polyclonal antibody (M19; Santa Cruz Biotechnology, Santa Cruz, CA, USA) (Clancy *et al*, 2003). Negative control slides omitted the primary antibody. In addition, embryonic neural tissue from wild-type (*EDD*^+/+^) and knockout (*EDD*^Δ/Δ^) mice (Saunders *et al*, 2004) was used as positive and negative controls, respectively.

Vascular endothelial growth factor (VEGF) staining was performed using an anti-VEGF monoclonal antibody (Ab-3, clone JH121; Neomarkers, Melbourne, VIC, Australia). Antigen retrieval was performed in 10 mM Tris/1 mM EDTA (pH 9.0) at 100°C for 20 min, followed by cooling at room temperature for 30 min. Antibody was used at 8 *μ*g ml^−1^ and incubated with the tissue sections overnight at 4°C. To visualise the antigen, sections were incubated with biotinylated goat anti-mouse secondary antibody (1 : 200), streptavidin–biotin–peroxidase complex (1 : 400) and DAB chromogen as described previously (Zeng *et al*, 2004). Immunohistochemistry staining for the cell cycle markers p53, p21^Wap/Cif1^, p27^Kip1^, cyclin D1 and cyclin E in this cohort has been reported previously (Bali *et al*, 2004).

Immunostaining was assessed by two independent observers (including a gynaecological pathologist JPS) blinded to patient stage and outcome and the discrepancies resolved by consensus. Expression of EDD was scored as the percentage of positive-staining nuclei (using assigned nominals: 0–25%=1; 26–50%=2; 51–75%=3; 76–100%=4) and intensity of staining (0–3), as described previously (Clancy *et al*, 2003). Initial analysis of the data showed that these parameters (% of positive nuclei and intensity) were correlated and therefore we multiplied the score for each sample to result in a scale of 0–12 indicative of the relative expression of EDD. Examination of the range of staining across the cohort determined a bimodal distribution around a median score of 6, which also reflected mean expression. We therefore defined a score of <6 as a low expression of EDD and ⩾6 as a high level of expression.

The percentage of cells and intensity of staining for cytoplasmic VEGF expression was similarly scored. As almost all of the carcinomas showed a high percentage of cells expressing VEGF (median expression 93%), we defined high expression as intensity of staining >2 based upon the median intensity of staining. Scoring and thresholds for the cell cycle markers p53, p21^Wap/Cif1^, p27^Kip1^, cyclin D1 and cyclin E in this cohort were as reported previously (Bali *et al*, 2004).

Correlations between gene expression and clinical and pathological variables were determined using the Mann–Whitney *U* test using Statview 4.5 software (Abacus Systems, Berkeley, CA, USA). A *P*-value of <0.05 was required for significance.

### Correlation of EDD expression to patient outcome

Clinical and pathological variables (FIGO stage, grade, age, menopausal status, optimal surgical debulking, chemotherapy regime, pre-operative CA 125 levels), gene expression (VEGF, p53 and p21^Wap/Cif1^) and EDD expression were correlated to patient outcome using comprehensive clinical follow-up data for each patient. Length of survival was defined from the date of initial diagnosis to the date of patient death or, in the case of surviving patients, their most recent follow-up date. For patients who exhibited a complete response to treatment (defined as no clinical, radiological or tumour marker evidence of disease for 3 months post-treatment), recurrence-free survival was measured from the date of diagnosis to the date of last follow-up or to disease recurrence (defined as either the reappearance of clinical symptoms by clinical examination or radiological investigation, or a rising serum CA125 level >35 U ml^−1^). Overall survival was defined as death due to ovarian cancer. Predictors of recurrence-free or overall survival were evaluated by univariate and multivariate analyses by Kaplan–Meier analysis and Cox proportional hazards models for dichotomised variables. Patients who had not experienced an event (ie disease recurrence or death due to ovarian cancer) at their most recent follow-up, or patients who were lost to follow-up, were censored. All *P*-values corresponded to two-sided tests, and *P*<0.05 was considered statistically significant. All statistical analyses were performed using Statview 4.5 software (Abacus Systems).

### Knockdown of EDD expression using siRNA

A2780/cp70 cells (Brown *et al*, 1993) were maintained in RPMI 1640 medium containing 10% FCS. For siRNA transfection, 5 × 10^5^ cells were seeded in 10 cm dishes approximately 16 h prior to transfection and then transfected with 2.8 pmol cm^−2^ of siRNA oligoribonucleotides using opti-MEM and Oligofectamine (Invitrogen Life Technologies, Mt Waverley, VIC, Australia). Annealed, HPLC and PAGE-purified siRNA oligoribonucleotides were obtained from Ambion (Austin, TX, USA): (EDD1) 5′-GCAGUGUUCCUGCCUUCUUdTdT-3′ and (EDD2) 5′-GCGACUCUCCAUGGUUUCUdTdT-3′. An siRNA directed against green fluorescent protein (GFP) 5′-CUGGAGUUGUCCCAAUUCUdTdT-3′ was used as a negative control. To confirm EDD knockdown, cells were harvested 48 h following transfection and whole lysates prepared and used for western blotting.

### Western blotting for EDD and CHK2 protein

SDS–PAGE and western blot analyses were performed according to standard protocols. Briefly, cell lysates were prepared in modified RIPA buffer (50 mM Tris HCl (pH 7.4), 1% (v/v) NP40, 0.5% (w/v) sodium deoxycholate, 0.1% (w/v) SDS, 137.5 mM NaCl, 1% (v/v) glycerol and 0.5 mM EDTA (pH 8.0) supplemented with protease inhibitors 1 mM sodium orthovanadate, 0.1 mg ml^−1^ aprotinin, 0.5 mg ml^−1^ leupeptin and 1 mM phenylmethylsulphonyl fluoride). To detect EDD protein, lysates (50 *μ*g) were electrophoresed on 6% polyacrylamide gels and transferred to PVDF membrane for 2.5 h. Membranes were blocked in 5% skim milk in Tris-buffered saline/0.1% Tween, followed by incubation in rabbit anti-EDD M19 antibody (1 : 10 000) or rabbit EDD1 antibody (1 : 10 000; Bethyl Laboratories, Montgomery, TX, USA) overnight at 4°C. Detection of *β*-actin (1 : 40 000 mouse anti-*β*-actin monoclonal antibody; Sigma Aldrich, Castle Hill, NSW, Australia) was used as a loading control. Following washing, membranes were incubated with horseradish peroxidase-conjugated rabbit or mouse anti-IgG antibody (1 : 10 000; Amersham Biosciences, Buckinghamshire, UK) for 1 h at room temperature. Detection of CHK2 protein using anti-CHK2 mouse monoclonal antibody (0.5 *μ*g ml^−1^; Upstate, Lake Placid, NY, USA) was performed according to the manufacturer's protocol. For all membranes, binding was detected using Western Lightning Chemoluminescence Reagent Plus (Perkin Elmer, Waltham, MA, USA) and quantified by densitometry.

### Colony-forming assays

Forty-eight hours following transfection with siRNA, 2–10 × 10^3^ A2780-cp70 cells replated in six-well plates were treated with 0–100 *μ*M cisplatin (*cis*-diaminodichloroplatinum; Sigma Aldrich) for 2 h. Following replacement of the cisplatin with fresh media, the plates were incubated for 6 days. Resulting colonies were stained using DiffQuik (Lab Aids, Ronkonkoma, NY, USA) and then scanned and quantified (ChemiDoc XRS and Quantity One 4.5.1; BioRad, Hercules, CA, USA). Results were expressed as the relative percentage of colonies as compared to untreated controls for each siRNA following adjustment for plating efficiency using untreated wells.

### Cisplatin sensitivity assays

To calculate IC_50_, cell lines were seeded into 96-well plates in a volume of 100  *μ*l at 0.5–2 × 10^3^ cells per well and incubated at 37°C overnight. On the following day, 50 *μ*l of complete medium containing cisplatin was added to duplicate wells to give a final drug concentration ranging from 625 to 20 *μ*M. Cell viability was determined using MTS assay (Promega, Annandale, NSW, Australia) on day 3 following drug treatment. Briefly, 100 *μ*l of PMS solution was mixed with 2 ml of MTS solution, and 20 *μ*l was added to each well. Following incubation at 37°C for 1 h, absorbance was read at 490 nm. IC_50_ values were calculated for each cell line from survival curves using data generated from between two and seven independent experiments. Correlation between EDD, CHK2 and cisplatin IC_50_ was determined using the Spearman rank correlation test using Statview 4.5 software (Abacus Systems).

## RESULTS

### Correlation of EDD staining with patient clinicopathological parameters

The clinicopathological treatment and outcome data of the patient cohort are shown in Table 1. A total of 151 patients diagnosed with serous epithelial ovarian carcinoma were examined. The majority of women were post-menopausal **(**77.3%) presenting with high (FIGO III–IV) stage (89.4%) serous ovarian carcinoma and treated with platinum-based chemotherapy (95.9%; Table 1). Approximately, 75% (*n*=114) of the patients had a complete response to surgery and adjuvant chemotherapy (defined as no clinical, radiological or tumour marker evidence of disease for 3 months post-treatment). The remaining patients (*n*=37, 25%) were classified as having progressive disease (no or partial response to surgery/chemotherapy, defined as a 50% reduction in tumour volume or CA 125 levels). The median follow-up time for the cohort was 35.5 months, with a median overall survival of 36.0 months. For patients with a complete response to treatment, the median time to relapse was 15.4 months following surgical diagnosis.

**Figure 1:**
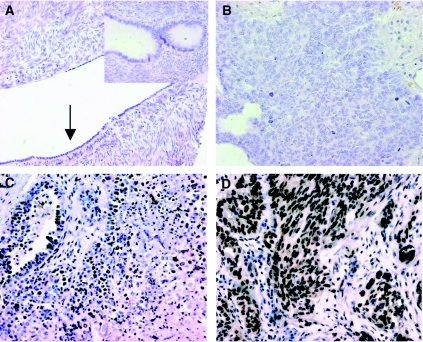
Representative IHC results for EDD expression in (**A**), normal ovary (ovarian surface epithelium arrowed) and ovarian inclusion cysts (inset); (**B**) negative EDD staining; (**C**) low (<6) EDD expression; and (**D**) high (⩾6) EDD expression in serous ovarian carcinomas. Original magnification × 20.

Expression of EDD protein was examined using IHC in archival paraffin-embedded fixed primary cancer tissue sampled prior to chemotherapy. The staining pattern of EDD was predominantly nuclear, concordant with its cellular location, with a low level of cytoplasmic staining in some cancers (Figure 1). EDD was expressed in almost all of the carcinomas examined (*n*=149/151, 98.7%**)**, with 57.6% (*n*=87/151) classified as having high nuclear expression of EDD (overall score ⩾6) (Table 1). EDD expression in undiseased (normal) ovarian surface epithelium or inclusion cysts, small foci of hyperplastic epithelium found within the cortex of normal ovaries and proposed as the site of origin of epithelial ovarian tumours (Auersperg *et al*, 2001), was rare (Figure 1). There was no association between EDD staining and any of the clinicopathological or gene expression variables tested, with the exception of preoperative serum CA125 levels (Table 2).

### EDD expression predicts disease recurrence and death

**Figure 2:**
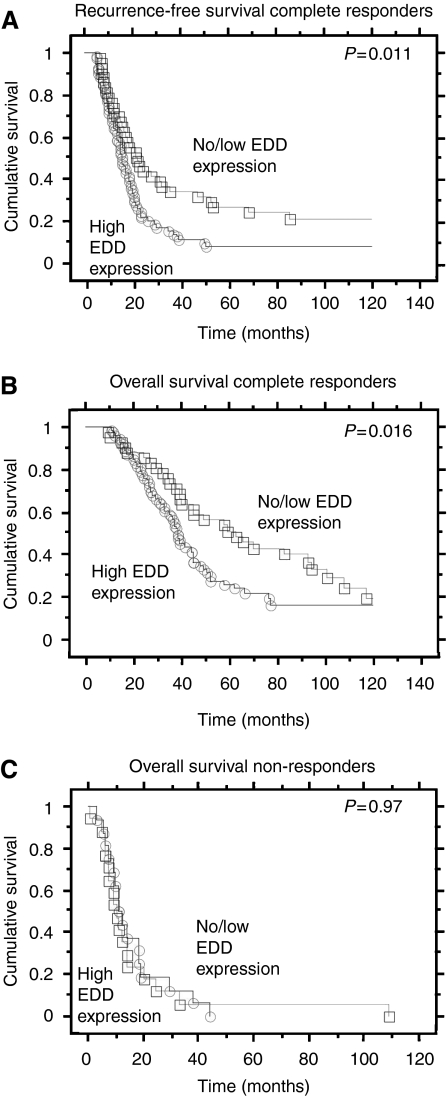
Association of EDD expression with ovarian cancer outcome. Kaplan–Meier survival curves and log-rank *P*-values showing (**A**) recurrence-free survival stratified by EDD expression (high *vs* low/absent) in serous ovarian cancer patients who suffered disease recurrence following initial complete response to treatment; (**B**) overall survival stratified by EDD expression (high *vs* low/absent) in serous ovarian cancer patients who suffered disease recurrence and death following initial complete response to treatment; (**C**) overall survival stratified by EDD expression (high *vs* low/absent) in patients with progressive disease (no or partial response to treatment).

Kaplan–Meier analysis and a Cox proportional hazards model were used to determine any associations between EDD expression and patient outcome in the cohort of 151 patients. We first examined the cohort of 114 patients who had initially responded to treatment (‘complete response’) and thus were susceptible to disease relapse. In this subgroup of patients, high FIGO stage (Cox proportional hazards ratio (HR) 3.50, *P*=0.002) and suboptimal surgical debulking (HR 2.59, *P*=<0.0001) were significantly associated with disease recurrence in univariate analysis (Table 3). Univariate analysis of EDD expression identified a significant association between EDD expression and disease recurrence (HR 1.75, *P*=0.011; Table 3) with a difference in median relapse time of 15.1 *vs* 17.3 months for patients with high and low EDD expression, respectively (Figure 2A). When combined in a multivariate model with FIGO stage and surgical debulking, the most important clinical prognostic indicator of patient outcome in ovarian cancer, EDD expression, remained a significant predictor of disease recurrence (HR 2.25, *P*=0.0004; Table 3). A similar association was found for high EDD expression and risk of death in this group of patients (HR 1.77, *P*=0.016), which was also independent of FIGO stage and surgical debulking (HR 1.96, *P*=0.006; Table 3), and resulted in a difference in median overall survival of 33.2 *vs* 42.5 months (Figure 2B). Moreover, if the survival analysis was restricted to patients with high stage (FIGO III/IV) and high tumour grade (grade 3) disease (*n*=69), high EDD expression was associated with an approximately four-fold increased risk of recurrence (HR 4.87, *P*<0.0001) and death (HR 4.02, *P*=0.003) following adjustment for surgical debulking.

We had previously shown that the cell cycle markers p53 and p21^Wap/Cif1^ were associated with disease recurrence in a subset of these patients (Bali *et al*, 2004). Only p21^Wap/Cif1^ retained its significant association with disease recurrence in this expanded cohort of patients (Table 3), and its incorporation into the multivariate survival model did not affect the ability of EDD to predict disease recurrence (HR 2.37, *P*=0.0006) and survival (HR 2.35, *P*=0.002).

There was no association of EDD expression with overall survival in patients (*n*=37) with progressive disease, that is those who failed to respond to chemotherapy (Figure 2C). Hence, the predictive effect of EDD expression on patient outcome may be limited to patients who initially respond to treatment.

### EDD expression is associated with resistance to cisplatin

As EDD plays a role in DNA damage response pathways, we next tested if the ability of EDD expression to predict disease recurrence, associated with the emergence of chemoresistance, was related to the type of adjuvant chemotherapy received. There was no association between EDD expression and response to different chemotherapeutic agents (Table 2) and neither did bivariate analysis of each treatment regime or individual chemotherapeutic agent (platinum, paclitaxel or alkylating agent (cyclophosphamide or melphalan) alter the ability of EDD to predict disease recurrence (data not shown). However, almost all of the patients received similar adjuvant chemotherapy (ie platinum-based therapy either alone or in combination), and none of the regimes were associated with an enhanced survival effect; hence, this cohort may not have sufficient power to perform these analyses.

**Figure 3:**
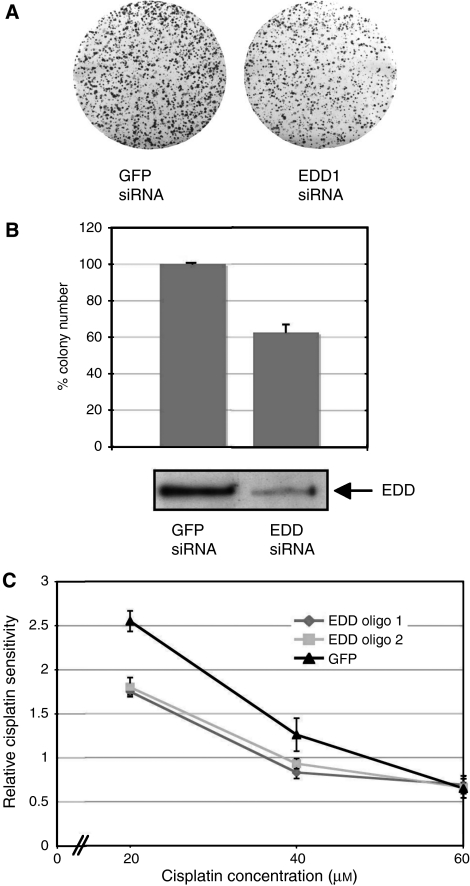
Knockdown of EDD expression using siRNA inhibits growth of cisplatin-resistant ovarian cancer cells. (**A**) Example of colony-forming assay showing inhibition of growth of ovarian A2780-cp70 cells transfected with siRNA against EDD (EDD siRNA1) as compared to siRNA against GFP (control) following treatment with cisplatin (20 *μ*M); (**B**) A2780-cp70 colony number following treatment with cisplatin (20 *μ*M) 48 h following transfection with siRNA against GFP (control) or EDD (EDD siRNA1). Results are expressed as the relative percentage of colonies as compared to untreated controls for each siRNA following adjustment for plating efficiency using untreated wells and are the means of duplicate experiments, each performed in triplicate wells. Confirmation of reduced EDD expression following siRNA transfection was determined by western blotting (below). (**C**) Dose–response curve of a representative experiment showing relative cisplatin sensitivity as determined by colony number of A2780-cp70 cells transfected with siRNA against EDD (EDD siRNA 1 and siRNA 2) compared to cells transfected with siRNA against GFP (control) after cisplatin treatment, as described above, and adjusted for untreated controls.

We, therefore, determined if EDD expression influences response to chemotherapy *in vitro*. Given that platinum compounds act via DNA damage pathways (Richardson and Kaye, 2005), we determined the effect of siRNA-mediated knockdown of EDD mRNA expression on the cisplatin-resistant ovarian cancer cell line A2780-cp70 following treatment with cisplatin. A2780-cp70 cells transfected with siRNA targeting EDD mRNA were approximately 40% more sensitive to cisplatin than those transfected with a control siRNA (GFP) (Figure 3A and B). This modified sensitivity to cisplatin was evident over a range of cisplatin concentrations and was reproducible with two separate siRNAs targeting different sequences of EDD mRNA (Figure 3C). Together, these data show that ovarian cancer cells expressing high levels of EDD are more resistant to cisplatin treatment than those with low EDD expression.

As our survival modelling suggested that the prognostic effect of EDD expression was limited to those patients who had a complete response to their treatment, we hypothesised that EDD expression may be associated with the acquisition of platinum resistance rather than intrinsic chemoresistance in serous ovarian cancer patients. To determine if there was a direct correlation between EDD expression and cisplatin resistance, we compared the relative cisplatin resistance (IC_50_) of six ovarian cancer cell lines of differing histological subtypes to their levels of EDD protein as determined by western blotting, as compared to the immortalised ovarian surface epithelial cell line HOSE 17.1 (Table 4). There was no correlation between EDD expression and cisplatin sensitivity (*r*_s_^2^=0.04, *P*=0.65). Moreover, EDD expression was lower in the cisplatin-resistant A2780-cp70 cell line than in its cisplatin-sensitive parent line A2780 (Table 4). These data suggest that cisplatin sensitivity is unlikely to be directly mediated by EDD but via cellular pathways in which it is involved. As CHK2 has been previously shown to be associated with cisplatin sensitivity in ovarian cancer (Zhang *et al*, 2005), we hypothesised that the modulation of cisplatin sensitivity by knockdown of EDD expression may be an indirect effect caused by reduced EDD binding to CHK2. We did not, however, find any association between EDD and CHK2 expression in these cell lines (*r*_s_^2^=0.19, *P*=0.34); however, CHK2 expression did not significantly correlate with cisplatin resistance in our experiments (*r*_s_^2^=0.49, *P*=0.11).

To determine if the effect of EDD on cisplatin sensitivity was potentially related to other functions of EDD other than DNA damage repair pathways, specifically proliferation and angiogenesis, we determined if EDD expression was correlated with cell cycle markers or VEGF expression as measured by IHC in this cohort of patients. There was no association between any of the cell cycle markers examined or VEGF expression with EDD (Table 2), suggesting that these functions of EDD are unlikely to be directly involved in the observed effect on cisplatin sensitivity.

## DISCUSSION

We have previously shown that EDD is specifically expressed in ovarian epithelial carcinoma but not in normal ovarian epithelial cells, which is most likely due to amplification at the *EDD* gene locus as a result of genomic instability (Clancy *et al*, 2003). In particular, high levels of EDD are expressed in the serous histological subtype of ovarian cancer. In the current study, we show that women exhibiting high EDD expression in their cancers at the time of diagnosis and those having a complete response to treatment (surgery and first-line chemotherapy) are approximately two-fold more likely to relapse and die than those with low EDD expression. This corresponded to a difference in median survival of greater than 9 months. The predictive effect of EDD expression on disease recurrence is independent of tumour stage or post-operative residual disease, the most important clinical prognostic indicators in serous ovarian cancer. Importantly, this correlated to an approximately four-fold increased risk of disease recurrence and death in patients with high stage, high-grade serous carcinoma. These cancers are the most common presentation of ovarian cancer and have a 5-year survival of approximately 20% (Ries *et al*, 2007), and there are very limited prognostic indicators in this group of patients (Birrer *et al*, 2007). This finding thus has important clinical implications and further implicates the molecular pathways in which EDD is involved as being important in serous ovarian cancer pathogenesis. Although our data arise from a large homogeneous cohort of well-characterised patients, these findings require validation in independent patient cohorts.

Relapsed ovarian cancer is often refractory to further cytotoxic treatment owing to the development of drug resistance. As in other cancers, the emergence of chemoresistance in ovarian cancer is linked to genes involved in DNA repair pathways, in particular to resistance to cytotoxic agents that act by causing DNA damage such as platinum compounds (Richardson and Kaye, 2005). For example, expression of *ERCC1* and *XPA*, components of the nucleotide excision repair pathways, are associated with platinum resistance in ovarian cancer (Damia *et al*, 1998; Selvakumaran *et al*, 2003); methylation and silencing of the mismatch repair gene *MLH1* in some ovarian cancers also result in loss of cisplatin sensitivity (Strathdee *et al*, 1999); and disruption of the FANC/BRCA pathway by methylation of *BRCA1* and *FANCF* alters sensitivity to cisplatin in ovarian cancer (Taniguchi *et al*, 2003). As one function of EDD is in the cellular response to DNA damage (Henderson *et al*, 2002, 2006; Honda *et al*, 2002; Munoz *et al*, 2007), we postulated that the ability of EDD to predict disease recurrence in serous ovarian cancer might similarly be related to the emergence of chemoresistance. The survival curves for those patients who initially responded to treatment were consistent with this hypothesis: all of the patients had a similar survival rate until approximately 12 months (disease recurrence) before diverging into two groups based on EDD expression with clearly different outcomes, suggestive of the emergence of chemoresistant cancer cells. Although we were unable to determine an association of EDD expression with response to chemotherapy, as almost all of the patients in our cohort received platinum-based treatment, we did, however, show that loss of EDD expression in a highly cisplatin-resistant ovarian cancer cell line is associated with enhanced sensitivity to cisplatin *in vitro*, thus supporting our hypothesis. Therefore, ovarian cancer cells with high levels of EDD may have an advantage in cell survival following chemotherapeutic attack by having a more efficient response to DNA damage and thus emerge as relapsed chemoresistant disease. We are currently undertaking experiments to determine if EDD expression can modulate the sensitivity to other classes of chemotherapy agents, including microtubule-targeting agents such as paclitaxel, also used in ovarian cancer.

Overexpression of DNA damage response proteins might be expected to give cells an advantage following exposure to cytotoxic drugs, such as platinum. For example, patients with non-functional (mutated) *BRCA1* or *BRCA2*, associated with hereditary breast and ovarian cancer, respond better to platinum chemotherapy owing to an increased sensitivity to DNA damage-induced cell death (Cass *et al*, 2003). Reintroduction of *BRCA1* into cells lacking functional BRCA1 enhances resistance to both radiation and paclitaxel (Zhou *et al*, 2003), and cells with higher than normal levels of BRCA1 protein are resistant to killing with platinum drugs (Husain *et al*, 1998). Similarly, ovarian cancers with high levels of EDD may be more likely to survive cytotoxic chemotherapy, for example, via the interaction between EDD and the critical DNA damage cell cycle checkpoint kinase CHK2 (Henderson *et al*, 2006). CHK2 phosphorylates a number of key proteins (including BRCA1) involved in various aspects of the DNA damage response including cell cycle arrest at the G2/M checkpoint, DNA repair and apoptosis (Motoyama and Naka, 2004). Degradation of CHK2 has been linked to cisplatin-induced resistance in ovarian cancer cells (Zhang *et al*, 2005), and alterations in the CHK2 pathway predict poor prognosis in ovarian cancer (Hashiguchi *et al*, 2004). We did not find an association between EDD and CHK2 expression in the ovarian cancer cell lines examined. However, we also did not find a correlation of CHK2 expression with cisplatin sensitivity, possibly reflecting the relatively small number of cell lines analysed, and thus it remains a possibility that the modulation of cisplatin sensitivity observed in our EDD knockdown experiments is an indirect effect caused by the interaction of EDD with CHK2.

Our survival data suggested that the prognostic ability of EDD may only be limited to patients who have a complete response to their treatment, and that EDD expression has no prognostic effect in women who are classified as being intrinsically resistant to chemotherapy. Despite clear criteria, classification of patients into complete and partial or non-responders to treatment remains subjective, which may influence these data. Indeed, our *in vitro* data did not support this hypothesis, as EDD expression was not directly correlated to cisplatin sensitivity, at least in ovarian cancer cell lines. Nonetheless, several gene expression profiling studies have identified increased EDD expression associated with chemoresistance in ovarian and other cancers. Increased EDD mRNA expression has been identified in post-chemotherapy carboplatinum-resistant serous ovarian cancer compared to post-chemotherapy sensitive carcinomas (Peters *et al*, 2005); in acquired cisplatin-resistant melanoma cell lines (Györffy *et al*, 2006); and in secondary brain tumours in acute lymphoblastic leukaemia patients arising following chemotherapy (Edick *et al*, 2005). Moreover, although not specifically attributed to EDD, gains at chromosome 8q12–23 are associated with development of platinum resistance in serous ovarian cancer (Wasenius *et al*, 1997). These independent observations support and extend the hypothesis that EDD overexpression is associated with acquired but not intrinsic chemoresistance in ovarian and other tumour types; however, this remains to be tested.

In addition to its role in cellular DNA repair, EDD has been implicated in other biological pathways that are suggestive of a functional link with ovarian cancer and that may additionally influence its prognostic power. First, EDD plays a pivotal role in the progesterone pathway, being both regulated by progestin and directly binding the progestin receptor and enhancing its transcriptional activity (Callaghan *et al*, 1998; Henderson *et al*, 2002). It has been known for several years that progesterone has a protective effect on ovarian cancer, the mechanism attributed to a reversal of the transformed phenotype including reduction of cyclin-dependent kinase activity and increased Fas/FasL-induced apoptosis (Blumenthal *et al*, 2003; Syed and Ho, 2003).

Secondly, we recently developed an *EDD* knockout mouse model that determined a functional role of EDD in vascular development and angiogenesis (Saunders *et al*, 2004). We predict that EDD overexpression in cancer may therefore enhance the ability of tumours to vascularise and spread. Marked neovascularisation is a feature of malignant ascites, the most common dissemination route of advanced serous ovarian cancer, and several angiogenic markers, including VEGF, are highly overexpressed in ovarian cancer (Ueda *et al*, 2005). We did not, however, find any association between VEGF and EDD expression in this cohort of patients, although such an association may become apparent in a larger patient sample.

E3 ubiquitin ligases have been implicated in cancer development via their regulation of key components of cellular pathways, including control of cell cycle progression (Burger and Seth, 2004). Similar to VEGF, we did not find any association between cell cycle markers and EDD expression in this cohort, including the tumour suppressor p53, which is a key mediator of cellular responses to DNA damage through modulation of cell cycle regulation, DNA repair and activation of pathways leading to apoptosis and is implicated in the development of cisplatin-resistance in ovarian cancer (Kigawa *et al*, 2001). Finally, although several ubiquitinylation targets of EDD have been identified, the contribution of its E3 ligase function in ovarian cancer is not yet known. Although somatic mutations in the *EDD* gene have been reported in gastric and colorectal tumours (Mori *et al*, 2002) and in mammary ductal carcinoma (Fuja *et al*, 2004), we have not identified any deleterious EDD mutations in a number of ovarian cancer cell lines (Clancy *et al*, 2003), and therefore, we predict that *EDD* mutation would be a very rare event in ovarian cancer and hence assume that *EDD* is functional in ovarian cancer.

In conclusion, although these findings remain to be validated in independent cohorts, we have identified EDD as a new molecular marker of prognosis in serous ovarian cancer, which may be related to its role in DNA damage repair pathways. Determining EDD expression levels may provide a clinically useful adjunct to current criteria in the management of serous ovarian cancer patients. Our observations that link EDD expression with platinum sensitivity merit further investigation in other large cohorts of serous ovarian cancer patients treated with adjuvant chemotherapy on a prospective basis, particularly in the context of a randomised treatment trial. Finally, E3 ubiquitin ligases have been classified as suitable targets for a novel class of anticancer drugs (Burger and Seth, 2004; Sun, 2006). Hence, EDD or DNA repair pathways involving EDD may have future application as novel therapeutic targets for patients who develop recurrent serous ovarian cancer.

## Figures and Tables

**Table 1:** Clinicopathological treatment, outcome and EDD expression data for the serous ovarian cancer patient cohort (*n*=151)^a^

**Variable**	***n* (%)**
*EDD expression*	
Low/absent (<6)	64 (42.4)
High (⩾6)	87 (57.6)
	
*Age (years)*	
>60	71 (47.0)
⩽60	80 (53.0)
	
*FIGO Stage*	
I, II	16 (10.6)
III, IV	135 (89.4)
	
*Tumour grade*	
1	7 (4.6)
2, 3	144 (95.4)
	
*Surgical debulking*	
Optimal ⩽1 cm	89 (58.9)
Suboptimal >1 cm	62 (41.1)
	
*Menopausal status (n=150)*	
Pre/peri	34 (22.7)
Post	116 (77.3)
	
*CA125 (n=128)*	
⩽500	54 (42.2)
>500	74 (57.8)
	
*Adjuvant chemotherapy* *(n=145)*	
P only	19 (13.1)
P+C	76 (52.5)
P+T	44 (30.3)
M only	6 (4.1)
	
*Outcome*	
Complete response to treatment	114 (75.5)
Progressive disease	37 (24.5)
Recurrence^b^	96 (84.2)
Death	123 (81.4)

C=cyclophosphamide; M=melphalan; P=platinum; T=paclitaxel.

a *n*=151 unless otherwise stated.

b In patients with complete response to treatment (*n*=114).

**Table 2:** Correlation of EDD expression with clinicopathological and gene expression variables^a^

**Variable**	***P*-value^b^**
Age	0.20
FIGO Stage	0.12
Tumour grade	0.17
Surgical debulking	0.59
Menopausal status	0.52
CA125	**0.027**
	
*Adjuvant chemotherapy*	
P only	0.94
P+C	0.44
P+T	0.38
M only	0.97
	
**VEGF** >2 *vs* ⩽2	0.80
**p53** >10% *vs* ⩽10%	0.50
**p27Kip1** ⩽65% *vs* >65%	0.22
**p21^Wap/Cif1^** ⩽10% *vs* >10%	0.22
**Cyclin D1** >10% *vs* ⩽10%	0.90
**Cyclin E** >10% *vs* ⩽10%	0.21

C=cyclophosphamide; M=melphalan; P=platinum; T=paclitaxel.

a EDD expression was modelled as a continuous variable. Clinicopathological and gene expression variables were dichotomised as shown in Table 1, as described in the text (VEGF) and Bali *et al*, 2004 (cell cycle markers).

b Bold type indicates significant *P*-values.

**Table 3:** A, univariate and B, multivariate Cox proportional hazards analyses of clinicopathological variables and gene expression with recurrence-free survival and overall survival in patients that exhibited a complete initial response to adjuvant chemotherapy

	**Recurrence-free survival**		**Overall survival**	
**Variable**	**HR (95% CI)**	***P*-value^a^**	**HR (95% CI)**	***P*-value**
(A)				
*Age (years)*				
⩽60 *vs* >60	0.95 (0.63–1.42)	0.80	0.79 (0.51–1.21)	0.28
				
*FIGO stage*				
III/IV *vs* I/II	3.50 (1.61–7.61)	**0.002**	4.48 (1.80–11.15)	**0.001**
				
*Tumour grade*				
2, 3 *vs* 1	2.54 (0.80–8.05)	0.11	2.55 (0.79–8.22)	0.12
				
*Surgical debulking*				
>1 cm *vs* ⩽1 cm	2.59 (1.69–3.97)	**<0.0001**	2.27 (1.46–3.52)	**0.0001**
				
*Menopausal status*				
Post *vs* pre/peri	1.14 (0.72–1.80)	0.59	1.61 (0.94–2.74)	0.08
				
*CA125*				
>500 *vs* ⩽500	1.42 (0.90–2.22)	0.13	1.08 (0.68–1.71)	0.76
				
*Adjuvant chemotherapy*				
P only N *vs* Y	1.38 (0.75–2.54)	0.29	1.14 (0.59–2.21)	0.70
P+C N *vs* Y	0.86 (0.57–1.29)	0.47	0.98 (0.64–1.51)	0.93
P+T N *vs* Y	0.90 (0.59–1.38)	0.63	0.93 (0.51–1.48)	0.76
M only N *vs* Y	1.95 (0.48–7.93)	0.35	1.25 (0.31–5.12)	0.76
				
*EDD expression*				
High *vs* low/absent	1.75 (1.14–2.69)	**0.011**	1.77 (1.11–2.80)	**0.016**
				
*VEGF expression*				
>2 *vs* ⩽2	1.39 (0.92–2.11)	0.12	1.22 (0.78–1.91)	0.38
				
*p53 expression*				
>10% *vs* ⩽10%	1.31 (0.84–2.05)	0.24	1.09 (0.68–1.73)	0.73
				
*p21* ^*Wap/Cif1*^				
<10% *vs* ⩾10%	1.96 (1.03–3.74)	**0.04**	2.30 (1.14–4.63)	**0.02**
				
(B)				
*FIGO Stage*				
III/IV *vs* I/II	3.84 (1.74–8.48)	**0.0009**	4.61 (1.82–11.66)	**0.001**
				
*Surgical debulking*				
>1 cm *vs* ⩽1 cm	2.60 (1.68–4.02)	**<0.0001**	1.92 (1.23–3.00)	**0.004**
				
*EDD expression*				
High *vs* low/absent	2.25 (1.44–3.52)	**0.0004**	1.96 (1.22–3.17)	**0.006**

C=cyclophosphamide; CI=confidence interval; HR=hazards ratio; M=melphalan; P=platinum; T, paclitaxel.

a Bold type indicates significant *P*-values.

**Table 4:** Correlation of relative EDD expression, relative CHK2 expression, and relative cisplatin sensitivity (IC_50_)^a^ of ovarian cancer cell lines^b^

**Cell line**	**Cisplatin IC_50_**	**EDD**	**CHK2**
HOSE17.1	0.90	1	1
CaOV3	1.20 (1)	0.30 (1)	3.78 (4)
OVCAR3	1.20 (1)	1.43 (5)	11.81 (6)
A2780	1.55 (3)	1.85 (6)	5.99 (5)
SKOV3	3.55 (4)	0.91 (4)	1.38 (1)
COLO316	11.20 (5)	0.53 (3)	3.43 (3)
A2780/cp70	13.60 (6)	0.37 (2)	2.44 (2)

a Determination of IC_50_ is described in the Materials and methods.

b Expression was determined by western blotting as described in the Materials and methods. Average expression was calculated from duplicate blots and is expressed as relative values as compared to HOSE17.1 cells. Ranks for the Spearman rank correlation test are shown in brackets.
