# The E3 ubiquitin ligase EDD is an adverse prognostic factor for serous epithelial ovarian cancer and modulates cisplatin resistance *in vitro*

**DOI:** 10.1038/sj.bjc.6604419

**Published:** 2008-05-27

**Authors:** P M O'Brien, M J Davies, J P Scurry, A N Smith, C A Barton, M J Henderson, D N Saunders, B S Gloss, K I Patterson, J L Clancy, V A Heinzelmann-Schwarz, R Murali, R A Scolyer, Y Zeng, E D Williams, L Scurr, A DeFazio, Di Quinn, C K W Watts, N F Hacker, S M Henshall, R L Sutherland

**Correction to:** *British Journal of Cancer* (2008) **98**, 1085–1093. doi:10.1038/sj.bjc.6604281

During revision and subsequent submission of the above paper to the journal, an author's name, Rajmohan Murali, was omitted from the authors list. The complete list of authors is given above.

